# Genetic evidence of gender difference in autism spectrum disorder supports the female-protective effect

**DOI:** 10.1038/s41398-020-0699-8

**Published:** 2020-01-15

**Authors:** Yi Zhang, Na Li, Chao Li, Ze Zhang, Huajing Teng, Yan Wang, Tingting Zhao, Leisheng Shi, Kun Zhang, Kun Xia, Jinchen Li, Zhongsheng Sun

**Affiliations:** 1 Institute of Genomic Medicine, Wenzhou Medical University, Wenzhou, Zhejiang 325025 China; 2 National Clinical Research Centre for Geriatric Disorders, Department of Geriatrics, Xiangya Hospital, Central South University, Changsha, Hunan 410008 China; 3 Beijing Institutes of Life Science, Chinese Academy of Science, Beijing, 100101 China; 4 Beijing Institute of Genomics, Chinese Academy of Sciences, Beijing, 100101 China; 5 Center for Medical Genetics, School of Life Sciences, Central South University, Changsha, Hunan 410008 China

**Keywords:** Medical genetics, Psychiatric disorders

## Abstract

Autism spectrum disorder (ASD) is a complex neurodevelopmental disorder with a male-to-female prevalence of 4:1. However, the genetic mechanisms underlying this gender difference remain unclear. Mutation burden analysis, a TADA model, and co-expression and functional network analyses were performed on de novo mutations (DNMs) and corresponding candidate genes. We found that the prevalence of putative functional DNMs (loss-of-function and predicted deleterious missense mutations) in females was significantly higher than that in males, suggesting that a higher genetic load was required in females to reach the threshold for a diagnosis. We then prioritized 174 candidate genes, including 60 shared genes, 91 male-specific genes, and 23 female-specific genes. All of the three subclasses of candidate genes were significantly more frequently co-expressed in female brains than male brains, suggesting that compensation effects of the deficiency of ASD candidate genes may be more likely in females. Nevertheless, the three subclasses of candidate genes were co-expressed with each other, suggesting a convergent functional network of male and female-specific genes. Our analysis of different aspects of genetic components provides suggestive evidence supporting the female-protective effect in ASD. Moreover, further study is needed to integrate neuronal and hormonal data to elucidate the underlying gender difference in ASD.

## Introduction

Autism spectrum disorder (ASD) represents a series of complex neurodevelopmental disabilities, characterized by deficits in social communication and restricted behaviors or interests^1^. In addition, it is characterized by a strong sexual dimorphism, as males are about four times more likely to be diagnosed with ASD than females^2^. Although this male prevalence is not unique to ASD, it has been regarded as an important clue toward uncovering the underlying etiology. Several plausible theories have been proposed to explain the increased risk of ASD in males^3^. Among these, the multiple threshold liability model has been most frequently discussed, which hypothesizes that multiple genetic factors contribute to the liability for developing ASD, and a higher threshold of genetic liability is required for females as compared with males; thus, this is also known as the “female protective model”^4–6^. This hypothesis has been supported by studies demonstrating that female cases have an excess of deleterious copy number variants, which ultimately disrupt more genes compared with those found in males^7–10^. The “extreme male brain theory” is another prominent hypothesis to explain that this gender bias, which suggests that fetal testosterone exposure may underlie gender difference in autistics traits^11^. Several studies have put forward evidence in favor of this theory as well^12–14^. In one study, steroidogenic activity was shown to be elevated during fetal development for males that were subsequently clinically diagnosed with ASD^14^, while another study found that females who had been exposed to high levels of testosterone in the womb had a more male-typical play style^13^.

Although the mechanism of this gender difference remains a mystery, progress in this regard is emerging from multiple aspects. With respect to clinical diagnosis, female patients with ASD showed a higher frequency of low intellectual level and greater internalizing symptoms compared with male patients with ASD^4,15,16^. In contrast, male patients with ASD showed greater social and externalizing behavioral problems, such as aggressive behaviors and increased repetitive stereotyped behaviors^4,15,17,18^. Structural neuroimaging studies further demonstrated certain frontal abnormalities in male patients with ASD that were absent in females^19,20^ along with a significant gender difference in the motor system and in areas that formed part of the “social brain”^21^. Moreover, the fetal testosterone level^12^ was found to be correlated with the gender difference in ASD, and androgens showed a male-bias prenatal influence over social brain circuitry^22^. Interestingly, a recent study indicated that differentially expressed genes in males with ASD were enriched in astrocyte and microglia markers^2^. In an animal model, a heterozygous *Chd8* mutation (N2373KfsX2) caused male-preponderant behavioral abnormalities in mice, suggesting its role in gender difference in ASD^23^.

Targeted sequencing^24–27^, whole-exome sequencing (WES)^28–34^, and whole-genome sequencing (WGS)^35,36^ have been successfully used by our group and others in detecting de novo mutations (DNMs) to prioritize risk genes for ASD. These coding DNMs have been estimated to contribute to 20–40% of ASD diagnoses^33,37,38^. In addition, integrated protein–protein interaction (PPI) and co-expression networks for ASD risk genes with functional DNMs indicate that risk genes are associated with biological processes related to Wnt signaling, chromatin remodeling, transcriptional regulation, and synaptic functions^39–44^. Moreover, we previously demonstrated that DNMs and functional network analysis could provide novel insights for comparing the convergences and divergences in different ASD subcategories^45^, for investigating the genetic mechanisms of brain size-related genes^46^ and vitamin-related genes^47^ in ASD, and for prioritizing novel candidate genes by integrating the genetic data of different neuropsychiatric disorders^48^.

Given the indispensable contribution of DNMs and the significant gender difference in ASD, the aim of this study was to decipher the genetic contribution underlying this gender difference based on integration of identified DNMs, candidate genes, co-expression, and functional networks in males and females diagnosed with ASD.

## Methods

### Data collection and annotation

Data of total 5748 trios and 1911 unaffected controls were collected from recent public trio-based WES/WGS studies^33–35,49^ on ASD (Supplemental Table S1). The patients in these studies were diagnosed with ASD using the gold standard Autism Diagnostic Observation Schedule (ADOS), the Autism Diagnostic Interview (ADI) and Autism Diagnostic Interview-Revised (ADI-R). Control samples were composed of unaffected SSC siblings. Only clinical information applied from SFARI base in the SSC was available. Age of the children diagnosed with ASD ranged from 4 to 18 years old, and we estimated the severity of ASD by IQ and restricted repetitive behaviors. Comprehensive annotation of each DNM was performed by ANNOVAR^50^ and VarCards^51^ with RefSeq (hg19, from UCSC) as described in our previous studies^45^, including (1) functional implications (e.g., gene region, effect, mRNA GenBank accession number, amino acid change, cytoband); (2) functional predictions for missense mutations; and (3) allele frequencies of different populations from various human genetic variation databases, including gnomAD, ExAC, ESP, and 1000G Genomes Project.

Only coding and splicing-site DNMs were selected for further analysis. In addition, DNMs with a minimum allele frequency > 0.1% in the public human genetic variation databases, mentioned above, were excluded. Deleterious missense mutations were predicted by the combination of REVEL^52^ and VEST3^53^ due to their best performance in predicting pathogenicity for missense variants^54^. We categorized deleterious missense DNMs and loss-of-functions (frameshift, stop-gain, stop-loss, splicing) DNMs as putative functional DNMs.

### Prioritization and classification of candidate genes

To prioritize the candidate DNMs, we divided the ASD subjects into three groups: all ASD subjects, female ASD subjects, and male ASD subjects. We then adopted the TADA^55^ model to prioritize candidate genes in the three groups, respectively. TADA is a weighted, statistical model integrating transmitted, de novo, case–control variants. Considering the accuracy and the amount of de novo genes, genes with a false discovery rate (FDR) < 0.2 were selected in further analysis. Finally, we combined all of the candidate genes and classified them into the following three subclasses: (1) genes with DNMs in both female and male patients, defined as shared genes; (2) genes with DNMs only in female patients, defined as female-specific genes; (3) and genes with DNMs only in male patients, defined as male-specific genes.

### Evaluation of the number of co-expressed genes

Developmental human brain RNA-seq data were curated from BrainSpan^56^, which contains expression data spanning different developmental stages, brain regions, gender, and age. Given the recognized importance of the prenatal stage in ASD development previously reported by us^45^ and other group^39,40^, expression data from fetal development stages between post-conception weeks 8 and 37 and in 15 brain regions were selected in further analysis, including 83 female-brain samples and 120 male-brain samples. We, respectively, calculated the Pearson correlation coefficients between any two candidate genes based on their expression levels in different male and female-brain regions. Gene pairs with |R| > 0.80 were regarded as being co-expressed according to our previous study^45^. We counted the number of other candidate genes that were co-expressed with the given gene in different male and female-brain regions. Finally, for each class of candidate gene (male-specific genes, female-specific genes, and shared genes), we employed pairwise Wilcoxon test to compare the number of co-expressed gene in all 15 male-brain regions and female-brain regions.

### Functional network analysis

Using the same developmental RNA-seq data from BrainSpan^56^, we calculated and assigned a Pearson correlation coefficient (R) for any two genes based on their expression levels in the different gender. Any gene pairs with |R| > 0.80 were classified as showing strong co-expression. Based on this criterion, we constructed a gene co-expression network with identical parameters to investigate the mechanisms behind the gender-differential risk. Any two genes within candidate genes that were co-expressed at the RNA level in female-brain samples and/or male-brain samples were connected to construct a functional network of candidate genes prioritized before.

Finally, to determine the specific functional relationship among sex-specific genes, Genes Ontology (GO) annotations were carried out using R software. Biological processes with *q*-value < 0.05 were considered to be statistically significantly enriched. Network diagrams were drawn by Cytoscape v.3.6.0 (https://cytoscape.org/)^57^.

## Results

### Increased mutation burden in female ASD patients

**Fig. 1:**
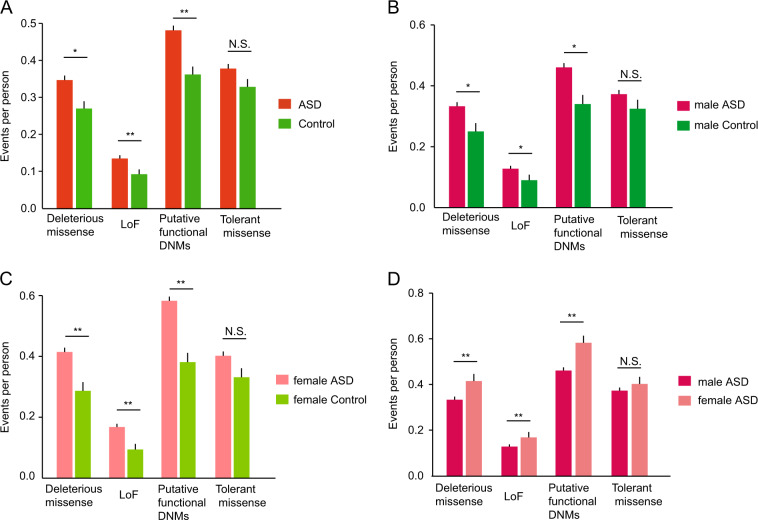
Mutation load of functional classes of DNMs in the coding region. **a** Mutation load per person in ASD versus control group. **b** Mutation load per person in male ASD subjects versus male controls. **c** Mutation load per person in female ASD subjects versus female controls. **d** Mutation load per person in male ASD subjects versus female ASD subjects. Mutation types are displayed by class. *p*-values were calculated by Fisher’s exact test. The “p.adjust” function in R was employed to calculate the corrected *p*-values for multiple comparisons, *adjusted *p* < 0.05, **adjusted *p* < 0.01, ***adjusted *p* < 0.001, N.S. not significant. The error bars represent 95% confidence intervals for the mean rates.

We collected DNMs from 5748 ASD trios (4783 male probands and 965 female probands) and 1911 control trios (900 unaffected male siblings and 1011 unaffected female siblings) from the ASC^34^, SSC^33^, MSSNG^35^, and other published studies^49^. Our analysis revealed that the probands carried significantly more loss-of-function (adjusted *p* *=* 2.28E-03, OR = 1.35) and deleterious missense mutations (adjusted *p* = 1.12E-02, OR = 1.19) than the matched controls, with no difference in tolerant missense mutations (adjusted *p* = 0.19, OR = 1.06, Fig. 1a; Supplementary Table S2).

In the gender-specific analysis, female ASD patients (adjusted *p* = 1.17E-03, OR = 1.44) and male ASD patients (adjusted *p* = 4.01E-02, OR = 1.25) were also found to harbor significantly more putative functional DNMs than the gender-matched controls (Fig. 1b, c; Supplementary Table S2). We further compared the DNM burden between female and male patients. Interestingly, there were significantly more loss-of-function (adjusted *p* = 8.09E-03, OR = 1.33, Fig. 1d) and deleterious missense mutations (adjusted *p* = 8.09E-03, OR = 1.26, Fig. 1d) in female ASD patients than in male ASD patients (Fig. 1d; Supplementary Table S2). We obtained a consistent result when combining deleterious missense and loss-of-function mutations (i.e., putative functional DNMs) (Fig. 1d). The mean number of putative functional DNMs was 0.46 and 0.59 in ASD male and female patients, respectively, representing an “ascertainment differential” of 0.59 − 0.46 = 0.13 (adjusted *p* = 5.48E-03, OR = 1.28, Fig. 1d; Supplementary Table S2). In addition, we observed a similar result in the SSC data set, suggesting that our stats were sufficiently powered. (Supplementary Fig. S1). However, as a negative control, there was no obvious difference from the perspective of tolerant missense mutations between female and male ASD patients (adjusted *p* *=* 0.16, OR = 1.09, Fig. 1d; Supplementary Table S2). Moreover, we did not find any significant difference in putative functional DNMs between female controls and male controls (Supplementary Table S2).

**Fig. 2:**
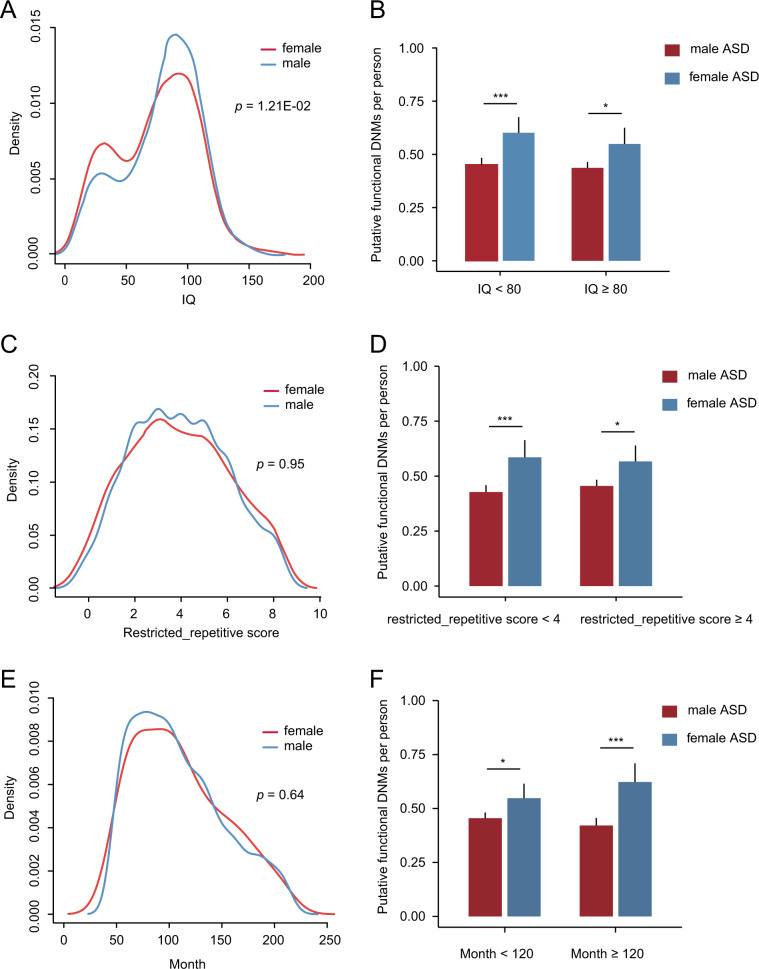
Association between putative functional DNMs and clinical phenotypes in the SSC data set. **a** The distribution of verbal IQ by gender. **b** Putative functional DNMs per person by gender in ASD patients with IQ < 80 and IQ ≥ 80. **c** The distribution of restricted repetitive score by gender. **d** Putative functional DNMs per person by gender in ASD patients with restricted repetitive score < 4 and restricted repetitive score ≥ 4. **e** The distribution of diagnostic month by gender. **f** Putative functional DNMs per person by gender in ASD patients with diagnostic month < 120 and diagnostic month ≥ 120. The *p*-values of comparison of distributions by gender were calculated by *t* test. The *p*-values of comparison of mutation burden by gender were calculated by Poisson test. *p < 0.05, **p *<* 0.01, ***p < 0.001, N.S. not significant.

Based on 2499 SSC samples (2162 female ASD patients and 337 male ASD patients) that the phenotypic data were available, we examined the association between mutation burden and IQ, restricted repetitive score as well as diagnostic age. Although there was a significant difference in IQ between female and male ASD patients (*p* = 1.21E-02, Fig. 2a), we still found a higher mutation burden of putative functional DNMs in female ASD patients than that in male ASD patients at different IQ (Fig. 2b). In addition, there was no significant difference in restricted repetitive score and diagnostic age between female and male ASD patients (Fig. 2c, e). However, affected females consistently showed a higher mutation burden of putative functional DNMs than males at different restricted repetitive score and diagnostic age (Fig. 2d, f). All these results indicated that affected females presented a higher mutation burden than affected males at different phenotypic conditions, providing a convincing evidence for “female protective model”.

### Prioritization of candidate genes

By employing TADA model, we ultimately prioritized 174 candidate genes (Table 1; Supplementary Table S3). These candidate genes were partitioned into three subclasses: (1) female-specific genes (*n* *=* *23*, genes with putative functional DNMs only existing in female samples); (2) male-specific genes (*n* = 91, genes with putative functional DNMs only existing in male samples); and (3) shared genes (*n* = 60, genes with putative functional DNMs existing in both female and male samples). Among these, 148 genes were listed in SFARI^58^ and/or AutismKB^59^ database. For example, one of the most significant candidate genes in the shared group, *SCN2A*, is an important autism-associated gene that is linked to voltage-gated sodium channel activity and ion channel activity^60^. Other shared genes, including *CHD2* and *PTEN*, listed in the “Syndromic” category of SFARI genes, are associated with dysregulation of estrogen dihydrotestosterone^22^. *KDM5B* is the most significant unique male-specific gene, which is related with chromatin organization and is associated with recessive developmental disorders^34,61,62^. *FOXP1* is another male-specific gene, which is related with androgen receptor signaling^63^. The female-specific gene *TCF4* is a reported autism-associated gene and is associated with coregulation of androgen receptor activity^64^. In addition, 26 genes were not included in the SFARI^58^ Gene or AutismKB^59^, such as *SPAG9*, *ITSN1*, and *MYPN*. Although some of these genes were not reported to be associated with gender difference in ASD, they may provide a reference for further study.

**Table 1:** Candidate genes of ASD based on TADA.

Class	Female-specific candidates (*n* = 23)	Male-specific candidates (*n* = 91)	Shared candidates (*n* = 60)
FDR < 0.001 (*n* = 17)	–	KDM5B	CHD8, SCN2A, SYNGAP1, ARID1B, PTEN, DYRK1A, ADNP, SLC6A1, SUV420H1, ANK2, SHANK3, TBR1, DSCAM, POGZ, CHD2, ITSN1
0.001 < FDR < 0.01 (*n* = 9)	–	SLC25A46, RANBP17, ASH1L	DNMT3A, GRIN2B, WDFY3, PRKAR1B, STXBP1, ASXL3
0.01 < FDR < 0.05 (*n* = 39)	AZGP1, ILF2, WAC, DDX3X, KIF11, UNC5B, SARM1, CALU	TDRD9, ASB14, TAF6, SET, PBX1, NUDT17, HYKK, APOA1BP, BSDC1, ZWILCH, USP45, SPAST, PPAN, PPAN-P2RY11, FOXP1, ZNF213, KMT2A, KDM6B, STXBP5L, LMTK3, CACNA2D3, SLC12A6, UBN2	NFE2L3, KATNAL2, SCN1A, TCF7L2, CNOT3, NCKAP1, KMT2C, RELN
0.05 < FDR < 0.1 (*n* = 42)	TCF4, DUS1L, GALNT18, RFX7, KIAA0100, PLXNB1, SRRM2	PHF2, DHX57, GIGYF1, PM20D1, RAI1, CSAD, TNC, SETBP1, KMT2E, OR10Z1, LRRK2, RIMS1, TNRC6B, RNF146, SHANK2, ZC3H4, PYHIN1, NXPE4, SLC4A9, LAMA3, TMEM39B, GLTSCR2	MYO1E, TRRAP, BCL11A, POM121, SMARCC2, MYT1L, OR8U1, KIF21A, PAPOLG, OR8U8, C18orf54, TBL1XR1, NLGN1
0.1 < FDR < 0.2 (*n* = 67)	RPS9, COL4A3BP, RASGRP3, RIPK1, GSAP, CBL, KCND3, MYPN	DIP2A, GABRB3, CDC23, TCF3, TSC2, CCIN, CCNT2, FBXO11, TLK2, CNGB3, UBE3C, ZC3H11A, NUAK1, LRRC4, RPH3A, MSH2, MYH10, SKI, DPP3, PSD3, RAPGEF4, TGM1, ERBB2IP, MTUS1, ATP1A1, GIGYF2, RBM19, RBP7, BRIP1, IRF2BPL, CASZ1, DENND5A, NUDCD2, FBXO18, SPAG9, SRGAP2B, KIAA0195, OR6C76, PRPF8, CHMP1A, PTK7, S100G	BTAF1, BRF1, FAM8A1, ACHE, OXR1, TSPYL1, MED13, TRIP12, AMPD1, CTNNB1, PLCD4, CTCF, GRIA2, SH2B1, CEP120, GRIK1, TPK1

Candidate genes with FDR < 0.2 were classified into three subclasses: (1) female-specific genes: genes with putative functional DNMs only in female patients; (2) male-specific genes: genes with putative functional DNMs only in male samples; (3) shared genes: genes with putative functional DNMs both in female and male patients

### Co-expression of three subclasses of candidate genes

**Fig. 3:**
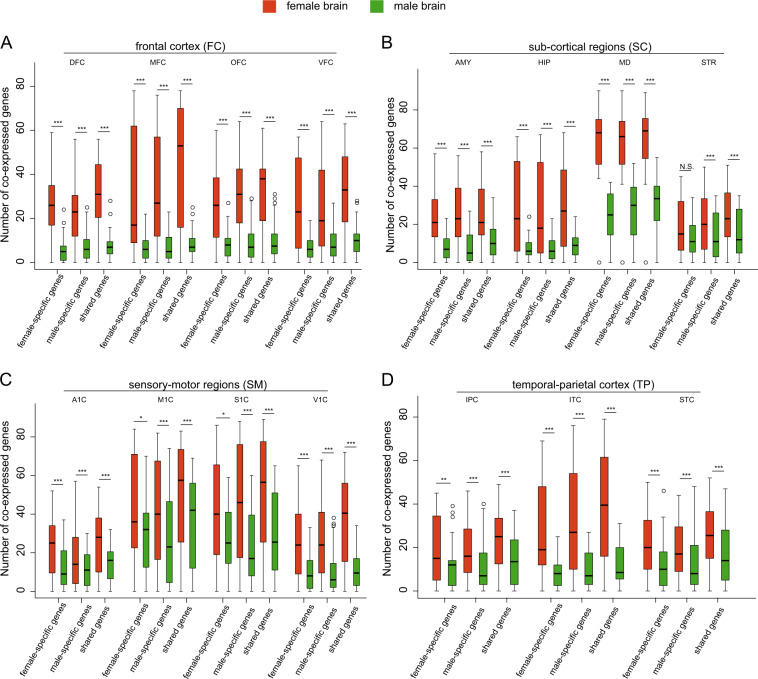
Co-expression in the three subclasses of candidate genes across gender. Comparisons of the number of co-expressed genes in the three subclasses of candidates genes across gender from (**a**) the FC subregion, including DFC (dorsolateral prefrontal cortex), MFC (anterior cingulate cortex), OFC (orbital frontal cortex), and VFC (ventrolateral prefrontal cortex); **b** the SC subregion, including AMY (amygdaloid complex), HIP (hippocampus), MD (mediodorsal nucleus of thalamus), and STR (striatum); **c** the SM subregion, including A1C (primary auditory cortex), M1C (primary motor cortex), S1C (primary somatosensory cortex), and V1C (primary visual cortex); **d** the TP subregion, including IPC (posteroinferior parietal cortex), ITC (inferolateral temporal cortex), and STC (posterior superior temporal cortex). *p*-values were calculated by the pairwise Wilcoxon test. *p < 0.05, **p< 0.01, ***p < 0.001, N.S. not significant.

Human brain development has close relation with expression pattern of relevant genes. Therefore, we performed the co-expression analysis with our candidate genes in 15 brain regions. As a result, we found all of the three subclasses of candidate genes being more frequently co-expressed in female-brain regions than in male-brain regions across multiple brain regions during prenatal development, which were reported as the most significant period associated with ASD by us^45^ and other group^39,40^, including the dorsolateral prefrontal cortex (DFC), anterior cingulate cortex (MFC), orbital frontal cortex (OFC), ventrolateral prefrontal cortex (VFC), amygdaloid complex (AMY), hippocampus (HIP), mediodorsal nucleus of thalamus (MD), striatum (STR), primary auditory cortex (A1C), primary motor cortex (M1C), primary somatosensory cortex (S1C), primary visual cortex (V1C), posteroinferior parietal cortex (IPC), inferolateral temporal cortex (ITC), posterior superior temporal cortex (STC), except for female-specific genes in the striatum (STR) region (Fig. 3). These data indicated that the deficiency of ASD risk genes may be more likely to be compensated by the greater amount of co-expressed genes in females, leading to lower prevalence in females.

### Functional analysis of candidate genes

To further investigate the specific functional pathways, we performed GO enrichment analysis for all the 174 candidate genes. Several biological processes known to be associated with ASD were enriched, including the Wnt signaling pathway (GO:0016055, *q*-value = 2.68E-02), axon development (GO:0061564, *q*-value = 2.34E-02), chromosome segregation (GO:0007059, *q*-value = 2.25E-02), negative regulation of neuron death (GO:1901215, *q*-value = 9.83E-06), and regulation of dendrite development (GO:0050773, *q*-value = 6.89E-03) (Supplementary Table S4). The genes in our three subclasses were randomly distributing in all functional blocks regardless of the specificity of genes, suggesting that sex-specific genes were more functionally convergent.

**Fig. 4:**
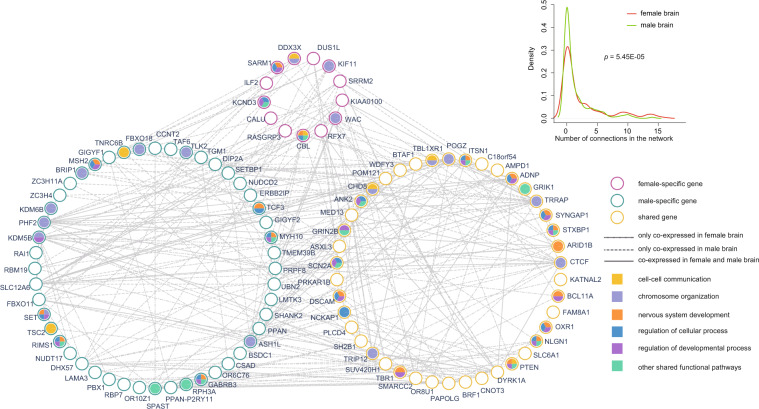
Functional network in sex-specific genes. Based on co-expression data from BrainSpan, 103 candidate genes formed a large interconnected functional network, mainly involving the following major functional blocks: cell–cell communication, chromosome organization, nervous system development, regulation of cellular process, and regulation of developmental process, distinguished by different fill colors of the nodes. The sex-specific genes are marked by different border colors of nodes. Different line types between nodes represent the interactions existing in female-brain samples or male-brain samples or in both female and male-brain samples. The top right image shows the distribution of co-expressed genes (genes with |R| > 0.80) among sex-specific genes in female and male-brain samples. *p*-values were calculated by the pairwise Wilcoxon test.

Furthermore, we connected all candidate genes found to be co-expressed at the mRNA level in female and/or male-brain samples to construct a functional network. This co-expressed network encompassed 103 genes and involved five main functional blocks: cell–cell communication (including two female-specific genes, two male-specific genes, and three shared genes), chromosome organization (including three female-specific genes, eight male-specific genes, and six shared genes), nervous system development (including two female-specific genes, six male-specific genes, and 11 shared genes), regulation of cellular process (including two female-specific genes, six male-specific genes, and nine shared genes), and regulation of developmental process (including two female-specific genes, six male-specific genes, and 13 shared genes). In addition, another 15 genes (including two female-specific genes, five male-specific genes, and eight shared genes) enriched in other shared biological processes (Fig. 4; Supplementary Table S4). We found that some of three subclasses of candidate genes were both enriched in the same functional blocks. For example, *WAC*, a female-specific gene, *ASH1L*, a male-specific gene and *TRIP12*, a shared gene, were all significantly enriched in the block of chromosome organization.

Meanwhile, we found that female-specific genes were co-expressed with 52 genes in female-brain samples, and only with 26 genes in male-brain samples. Male-specific genes were co-expressed with 135 genes in female-brain samples, and only with 94 genes in male-brain samples. Similarly, 153 genes were co-expressed with shared genes in female-brain samples, and 83 genes were co-expressed with shared genes in male-brain samples (Fig. 4). It again suggested that three subclasses of candidate genes were significantly more frequently co-expressed in female-brain samples.

## Discussion

With the development of targeted sequencing^24–27^, WES^28–34^, and WGS^35,36^ methodologies, DNMs of ASD have recently been identified, and risk genes have been prioritized, providing novel insight into the pathogenic factors contributing to the susceptibility and development of ASD. However, pathogenesis underlying the male prevalence of the disorder has thus far remained unclear. Through integrated analysis of DNMs of ASD, transcriptome data, and construction of gender-specific and overlapping co-expression networks, our results suggest that female ASD patients harbor more putative functional (de novo loss-of-function and deleterious missense mutations) DNMs than male ASD patients, leading to more serious clinical phenotypes in females. Although this is in line with a previous study demonstrating a significantly higher burden of deleterious copy number variations in females with ASD compared with males^8^, our analysis is more comprehensive and detailed. Thus, our findings provide convincing evidence to further support the “female protective model” in ASD that posits that females need a higher minimum threshold to manifest the ASD phenotype as compared with males^4,6^. This finding is in line with that higher mutation burden results in a more severe clinical phenotype in females. In addition, these results suggest that it is more difficult for clinicians to diagnose in females than males. We would encourage that the different diagnostic criteria for females and males could be used in the clinicals. Sex-specific thresholds may be more helpful for ASD screening and diagnosis. However, we are not implying that this hypothesis can account completely for gender difference in ASD. Instead, due to lack of the data of sex steroid hormones, we could not exclude “extreme male brain”^11,22^. Thus, we propose for examining females with the levels of multiple sex steroid hormones during pregnancy.

We also considered about the role of hemizygous LoF variants on the X chromosome in male ASD patients. A previous study performed an analysis in 993 cases and 869 controls, and estimated a ~2% contribution to ASD risk in males^65^. Based on exome data of quad-samples from SSC, we identified 69 and 23 X-linked LoF variants in 1571 male probands and 847 male siblings, respectively. Based on the ascertainment differentials between male probands and male siblings (0.044 versus 0.027), these data predicted a contribution of X-linked hemizygous LoF variants to ~1.7% of ASD cases. All these data showed that hemizygous LoF variants do contribute to male ASD, but only explain a small proportion of the male gender bias observed in ASD.

It is to be noted that some studies indicated that intellectual level and behavioral phenotypes might influence gender difference in ASD^16^. In addition, possible factors in diagnostic processed, such as biases in diagnostic patterns of clinician^66^, age of diagnosis^67^, the phenomena reflecting gender-based interpretation bias from sources of referral or diagnostician^68^, might influence the sample and some conclusions. Further studies need to combine these factors with genetic factor to understand the gender bias in ASD. Our control samples made up of unaffected siblings (US) of children with ASD may have some overlapping phenotypes with ASD. Previous study indicated that the US group was indistinguishable from typically developmental (TD) group at the behavioral level and similar neural signatures in trait activity between the US and ASD groups^69^. The control samples in this study were made up of unaffected siblings (US) of patients with ASD, and the US may have a high level of autistic traits. We speculate that it may show a more significant gender difference in mutation burden analysis if control samples are from TD children. In addition, we could only have access to the clinical phenotypes of SSC data set. Although our conclusion is sufficiently powered, we encourage a larger sample size and detailed clinical phenotype can be employed in the future study.

Based on putative functional DNMs, we further prioritized 174 candidate genes with a TADA model and identified sex-specific genes, including 23 female-specific genes, 91 male-specific genes, and 60 shared genes. Considering the overall low frequency of DNMs and the limit of sample size, the sex specificity of these three subclasses of candidate genes needs to be validated in a larger data set. Nevertheless, the three subclasses of candidate genes identified in our study can functionally reflect the gender bias to some extent. For example, *FOXP1*, a male-specific gene, has been reported to influenced by estrogen dihydrotestosterone dysregulation that acts via androgen receptor to influence gene expression in human neural stem cells^22^, relevant to the hypothesis that sex hormones may function as male-specific risk factors or female-specific protective factors^4,11,14^. *TCF4*, a female-specific gene, has been revealed to play an important role in nervous system development^70^ and participate in the coregulation of androgen receptor activity^64^, possibly associated with the theory of “Extreme male brain”.

We provided a new perspective of analysis at the co-expression in the gender difference of ASD and demonstrated that female-specific genes, male-specific genes, and shared genes were more frequently co-expressed in female-brain samples, suggesting that the deficiency of ASD candidate genes may be more likely to be compensated by the greater amount of co-expressed genes in females than in males, resulting in high prevalence in males. These data further bring evidence for supporting “female protective effect”. Moreover, all of three subclasses of candidate genes were co-expressed with each other and enriched in the same biological process, suggesting that a convergent functional network of sex-specific genes. Although there was no sex-specific biological process identified in our network, it may imply that gender difference could not be completely explained by DNMs and may be involved in biological network, systems biological, expression levels, and other aspects.

In summary, our results provide convincing evidence for the “female protective effect” to explain the gender bias in ASD from the aspects of DNMs, gene expression levels, and the functional network. This result further implies that DNMs can only explain a small part of the gender difference in ASD, and the detailed mechanisms are clearly more complex. Therefore, future work investigating the gender difference in ASD is needed to integrate distinct aspects that are typically considered in isolation, such as inherited variants^71^, epigenetic factors^72^, structural variants^73^, environmental factors^74,75^, and mRNA levels^2^. Moreover, we identified novel candidate genes that might reveal specific functions related to the gender difference in ASD, which can offer guidance for further research to provide new insight into clinical diagnoses and treatments.

## Supplementary information

Table S1

Table S2

Figure S1

Table S3

Table S4

Supplemental information
