# Supplementary material for: Genetic evidence of gender difference in autism spectrum disorder supports the female-protective effect
Source: Transl Psychiatry. 2020 Jan 15;10:4. doi: 10.1038/s41398-020-0699-8 (PMC7026157; doi:10.1038/s41398-020-0699-8)
Supplement: Supplementary file 1 — Table S1 [file 41398_2020_699_MOESM1_ESM.docx]

**Supplemental Table S1. Literature information of cataloged DNMs in this study.**

| **Reference** | **Diagnostic standard** | **Disease** | **Trios** | **Male** | **Female** | **DNMs** | **Exonic DNMs** |
| --- | --- | --- | --- | --- | --- | --- | --- |
| Ivan Iossifov et al. Nature 2014 | ADOS, ADI-R | ASD | 2,508 | 2,167 | 341 | 3,397 | 2,723 |
| Silvia De Rubeis et al. Nature 2014 | ADOS, ADI | ASD | 1,445 | 1,204 | 241 | 1,702 | 1,684 |
| Ryan K C Yuen et al. Nature Medicine 2015 | ADOS, ADI-R | ASD | 170 | 141 | 29 | 15,511 | 203 |
| C Yuen RK et al. Nature Neuroscience 2017 | ADOS, ADI-R | ASD | 1,625 | 1,271 | 354 | 140,408 | 1,972 |
| Subtotal of ASD | - | ASD | 5,748 | 4,783 | 965 | 161,018 | 6,582 |
| Ivan Iossifov et al. Nature 2014 | - | Control | 1,911 | 900 | 1,011 | 2,285 | 1,832 |

Note: Autism Diagnostic Observation Schedule (ADOS); Autism Diagnostic Interview (ADI); Autism Diagnostic Interview-Revised (ADI-R).
