# Supplementary material for: Genetic evidence of gender difference in autism spectrum disorder supports the female-protective effect
Source: Transl Psychiatry. 2020 Jan 15;10:4. doi: 10.1038/s41398-020-0699-8 (PMC7026157; doi:10.1038/s41398-020-0699-8)
Supplement: Supplementary file 2 — Table S2 [file 41398_2020_699_MOESM2_ESM.docx]

**Supplemental Table S2.** **Odds ratio of functional classes of DNMs in coding region.**

|  | **Trios** | **Deleterious missense** | **LoF** | **Tolerant missense** | **Putative functional DNMs** |
| --- | --- | --- | --- | --- | --- |
| ASD | 5,748 | 1,992 | 773 | 2,170 | 2,765 |
| ASD male | 4,783 | 1,592 | 611 | 1,782 | 2,203 |
| ASD female | 965 | 400 | 162 | 388 | 562 |
| Control | 1,911 | 515 | 176 | 627 | 691 |
| Control male | 900 | 225 | 81 | 292 | 306 |
| Control female | 1,011 | 290 | 95 | 335 | 385 |
| ASD vs Control | OR | 1.19 | 1.35 | 1.06 | 1.23 |
|  | 95% Cl | 1.05-Inf | 1.14-Inf | 0.95-Inf | 1.10-Inf |
|  | P-value | 8.43E-03 | 1.14E-03 | 0.19 | 1.13E-03 |
|  | Adjusted | **1.12E-02** | **2.28E-03** | 0.19 | **2.28E-03** |
| ASD male vs ASD female | OR | 1.26 | 1.33 | 1.09 | 1.28 |
|  | 95% Cl | 1.08-Inf | 1.10-Inf | 0.94-Inf | 1.12-Inf |
|  | P-value | 4.33E-03 | 6.07E-03 | 0.16 | 1.37E-03 |
|  | Adjusted | **8.09E-03** | **8.09E-03** | 0.16 | **5.48E-03** |
| Control male vs Control female | OR | 1.14 | 1.04 | 1.01 | 1.11 |
|  | 95% Cl | 0.92-Inf | 0.76-Inf | 0.82-Inf | 0.91-Inf |
|  | P-value | 0.17 | 0.46 | 0.48 | 0.20 |
|  | Adjusted | 0.40 | 0.48 | 0.48 | 0.40 |
| ASD male vs Control male | OR | 1.22 | 1.30 | 1.06 | 1.25 |
|  | 95% Cl | 1.03-Inf | 1.03-Inf | 0.90-Inf | 1.06-Inf |
|  | P-value | 2.57E-02 | 3.01E-02 | 0.30 | 1.15E-02 |
|  | Adjusted | **4.01E-02** | **4.01E-02** | 0.30 | **4.01E-02** |
| ASD female vs control female | OR | 1.36 | 1.68 | 1.14 | 1.44 |
|  | 95% Cl | 1.11-Inf | 1.28-Inf | 0.94-Inf | 1.19-Inf |
|  | P-value | 5.20E-03 | 5.45E-04 | 0.14 | 5.87E-04 |
|  | Adjusted | **6.93E-03** | **1.17E-03** | 0.14 | **1.17E-03** |

In each group, exonic DNMs were classified into different functional classes. Compared to control group, P-values were calculated on the basis of Fisher’s exact test. We referred to LoF/deleterious SNVs as damaging SNVs. The rare LoF/deleterious SNVs and rare frame-shift indels, which were regarded as possible functional mutations. P-values below 0.05 are highlighted in bold. Deleterious missense mutations were predicted by the combine of REVEL and VEST3 (score > 0.5).
