# Supplementary figures and images for: Genetic evidence of gender difference in autism spectrum disorder supports the female-protective effect
Source: Transl Psychiatry. 2020 Jan 15;10:4. doi: 10.1038/s41398-020-0699-8 (PMC7026157; doi:10.1038/s41398-020-0699-8)

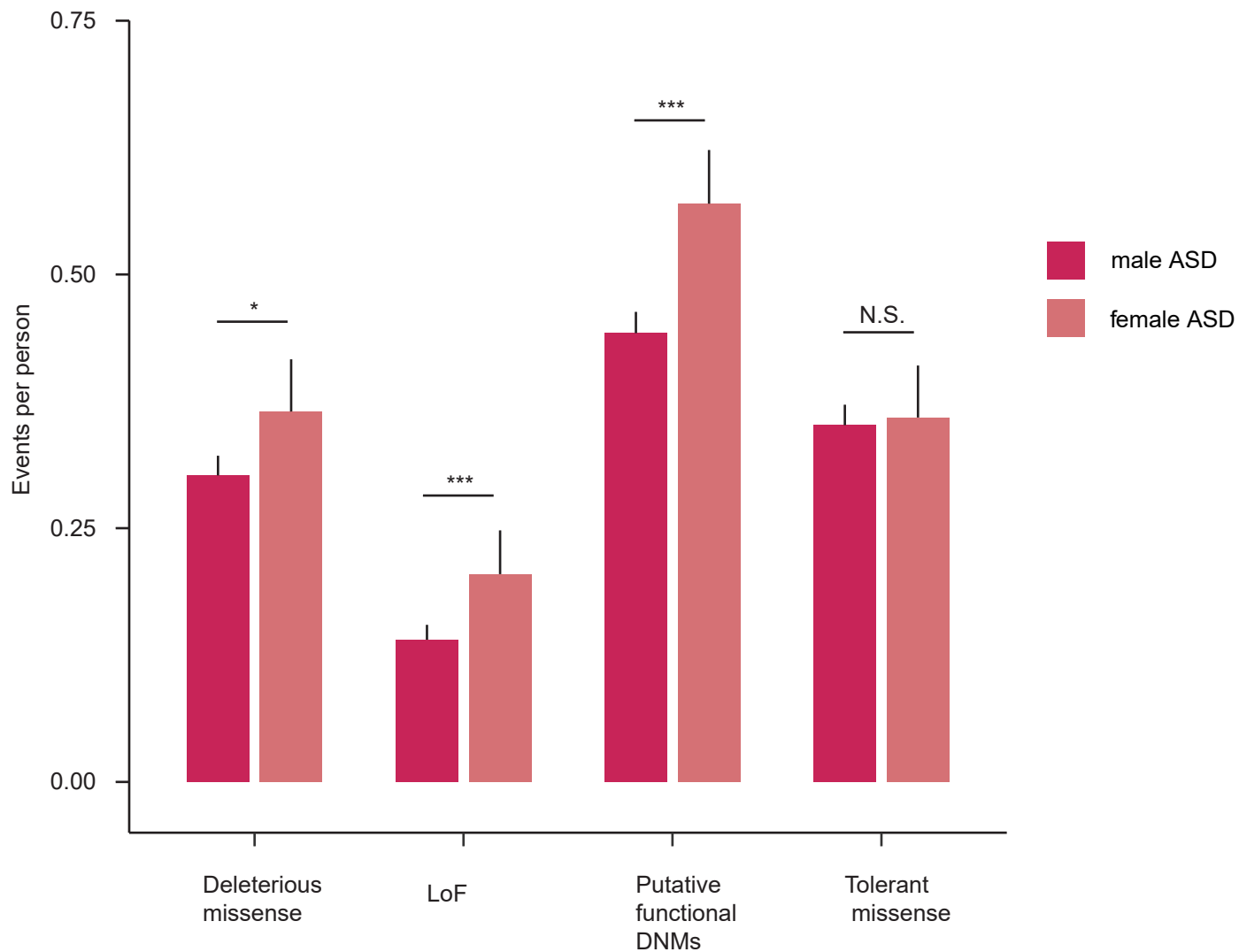

Supplement: Supplementary file 3 — Figure S1 [file 41398_2020_699_MOESM3_ESM.pdf]
