# Supplementary material for: Genetic evidence of gender difference in autism spectrum disorder supports the female-protective effect
Source: Transl Psychiatry. 2020 Jan 15;10:4. doi: 10.1038/s41398-020-0699-8 (PMC7026157; doi:10.1038/s41398-020-0699-8)
Supplement: Supplementary file 5 — Table S4 [file 41398_2020_699_MOESM5_ESM.docx]

**Supplemental Table S4. Shared GO terms in the three subclasses of candidate genes.**

| **Functional blocks** | **Go terms** | **Observed in female-specific genes** | **Observed in male-specific genes** | **Observed in shared gene** | **Q value** |
| --- | --- | --- | --- | --- | --- |
| cell-cell communication | cell-cell signaling by wnt (GO:0198738) | 1 | 6 | 5 | 2.70E-2 |
|  | regulation of phosphatidylinositol 3-kinase signaling (GO:0014066) | 3 | 1 | 2 | 4.30E-02 |
|  | Wnt signaling pathway (GO:0016055) | 1 | 6 | 5 | 2.68E-02 |
| chromosome organization | chromosome segregation (GO:0007059) | 2 | 3 | 5 | 2.25E-02 |
|  | DNA conformation change (GO:0071103) | 1 | 5 | 4 | 9.57E-03 |
|  | DNA duplex unwinding (GO:0032508) | 1 | 2 | 2 | 1.81E-02 |
|  | DNA geometric change (GO:0032392) | 1 | 2 | 2 | 2.51E-02 |
|  | histone modification (GO:0016570) | 1 | 6 | 5 | 1.76E-02 |
| nervous system development | axon development (GO:0061564) | 2 | 2 | 8 | 2.34E-02 |
|  | Axonogenesis (GO:0007409) | 2 | 2 | 8 | 3.40E-02 |
|  | dendrite development (GO:0016358) | 1 | 1 | 8 | 1.20E-03 |
|  | dendrite morphogenesis (GO:0048813) | 1 | 1 | 5 | 5.88E-03 |
|  | positive regulation of nervous system development (GO:0051962) | 2 | 4 | 6 | 2.25E-02 |
|  | positive regulation of neurogenesis (GO:0050769) | 2 | 4 | 6 | 8.77E-03 |
|  | positive regulation of neuron differentiation (GO:0045666) | 2 | 4 | 5 | 6.89E-03 |
|  | positive regulation of neuron projection development (GO:0010976) | 1 | 2 | 5 | 3.18E-02 |
|  | regulation of neuron death (GO:1901214) | 3 | 5 | 6 | 3.64E-05 |
|  | regulation of neuron projection development (GO:0010975) | 2 | 3 | 9 | 1.42E-03 |
| regulation of cellular process | actin filament-based movement (GO:0030048) | 1 | 2 | 3 | 2.25E-02 |
|  | negative regulation of neuron apoptotic process (GO:0043524) | 1 | 3 | 5 | 4.68E-04 |
|  | negative regulation of neuron death (GO:1901215) | 2 | 5 | 6 | 9.83E-06 |
|  | neuron apoptotic process (GO:0051402) | 1 | 3 | 7 | 5.26E-04 |
|  | neuron death (GO:0070997) | 3 | 5 | 7 | 3.06E-05 |
|  | positive regulation of cell development (GO:0010720) | 2 | 4 | 6 | 2.25E-02 |
|  | positive regulation of cell projection organization (GO:0031346) | 1 | 2 | 7 | 1.88E-02 |
| regulation of developmental process | developmental growth involved in morphogenesis (GO:0060560) | 1 | 4 | 3 | 1.77E-02 |
|  | regulation of cell morphogenesis (GO:0022604) | 2 | 3 | 9 | 2.18E-03 |
|  | regulation of cell morphogenesis involved in differentiation (GO:0010769) | 2 | 8 | 1 | 1.42E-03 |
|  | regulation of dendrite development (GO:0050773) | 1 | 1 | 5 | 6.89E-03 |
|  | regulation of dendrite morphogenesis (GO:0048814) | 1 | 1 | 4 | 3.95E-03 |
|  | regulation of neuron apoptotic process (GO:0043523) | 1 | 3 | 6 | 1.10E-03 |
|  | regulation of oxidative stress-induced cell death (GO:1903201) | 1 | 1 | 2 | 4.08E-02 |
|  | regulation of transmembrane transport (GO:0034762) | 2 | 2 | 7 | 3.40E-02 |
| Other shared functional pathways | cardiac muscle cell action potential involved in contraction (GO:0086002) | 1 | 1 | 2 | 2.41E-02 |
|  | cardiac muscle cell contraction (GO:0086003) | 1 | 1 | 2 | 4.08E-02 |
|  | establishment of organelle localization (GO:0051656) | 1 | 7 | 4 | 1.58E-02 |
|  | organelle localization (GO:0051640) | 1 | 7 | 6 | 6.89E-03 |
|  | regulation of membrane potential (GO:0042391) | 1 | 5 | 10 | 5.61E-05 |
