# Supplementary material for: Genetic evidence of gender difference in autism spectrum disorder supports the female-protective effect
Source: Transl Psychiatry. 2020 Jan 15;10:4. doi: 10.1038/s41398-020-0699-8 (PMC7026157; doi:10.1038/s41398-020-0699-8)
Supplement: Supplementary file 6 — Supplemental information [file 41398_2020_699_MOESM6_ESM.docx]

**Supplemental figure legend**

**Figure S1. Mutation load of functional classes of DNMs in the SSC.** Mutation load per person in male ASD subject versus female ASD subjects. Mutation types are displayed by class. P-values were calculated by Poisson test. The “p.adjust” function in R was employed to calculate the corrected p-values for multiple comparisons, * adjusted *P* < 0.05, ** adjusted *P* < 0.01, *** adjusted *P* < 0.001, N.S., not significant. The error bars represent 95% confidence intervals for the mean rates.
